# Effects of asenapine on depressive symptoms in patients with bipolar I disorder experiencing acute manic or mixed episodes: a post hoc analysis of two 3-week clinical trials

**DOI:** 10.1186/1471-244X-11-101

**Published:** 2011-06-20

**Authors:** Armin Szegedi, Jun Zhao, Arjen van Willigenburg, Kari R Nations, Mary Mackle, John Panagides

**Affiliations:** 1 Merck Research Laboratories, Rahway, NJ, USA; 2 At Time of Research: Schering-Plough (formerly Organon), Roseland, NJ, USA; 3 At Time of Research: Schering-Plough (formerly Organon), now Merck Research Laboratories, Summit, NJ, USA

**Keywords:** asenapine, bipolar I disorder, depressive symptoms, post hoc analysis

## Abstract

**Background:**

Asenapine demonstrated superiority over placebo for mania in bipolar I disorder patients experiencing acute current manic or mixed episodes in 2 randomized, placebo-and olanzapine-controlled trials. We report the results of exploratory pooled post hoc analyses from these trials evaluating asenapine's effects on depressive symptoms in patients from these trials with significant baseline depressive symptoms.

**Methods:**

In the original trials (A7501004 [NCT00159744], A7501005 [NCT00159796]), 977 patients were randomized to flexible-dose sublingual asenapine (10 mg twice daily on day 1; 5 or 10 mg twice daily thereafter), placebo, or oral olanzapine 5-20 mg once daily for 3 weeks. Three populations were defined using baseline depressive symptoms: (1) Montgomery-Asberg Depression Rating Scale (MADRS) total score ≥20 (n = 132); (2) Clinical Global Impression for Bipolar Disorder-Depression (CGI-BP-D) scale severity score ≥4 (n = 170); (3) diagnosis of mixed episodes (n = 302) by investigative site screening. For each population, asenapine and olanzapine were independently compared with placebo using least squares mean change from baseline on depressive symptom measures.

**Results:**

Decreases in MADRS total score were statistically greater with asenapine versus placebo at days 7 and 21 in all populations; differences between olanzapine and placebo were not significant. Decreases in CGI-BP-D score were significantly greater with asenapine versus placebo at day 7 in all categories and day 21 in population 1; CGI-BP-D score reductions were significantly greater with olanzapine versus placebo at day 21 in population 1 and day 7 in populations 2 and 3.

**Conclusions:**

These post hoc analyses show that asenapine reduced depressive symptoms in bipolar I disorder patients experiencing acute manic or mixed episodes with clinically relevant depressive symptoms at baseline; olanzapine results appeared to be less consistent. Controlled studies of asenapine in patients with acute bipolar depression are necessary to confirm the generalizability of these findings.

## Background

Bipolar disorder is a serious chronic medical condition that typically is cyclical, characterized by manic/hypomanic, depressed, or mixed states, and associated with a high risk for suicide [1,2]. Although manic episodes are considered the hallmark state of bipolar I disorder, patients spend up to 4 times more symptomatic time in depressed states [3], and it is depression that primarily contributes to functional disability and high rates of suicide [4-6]. In 2001, the World Health Organization reported that bipolar affective disorders rank within the top 10 causes of disability among all medical conditions, as measured in years lived with disability [7].

Although a number of treatment options have been established for acute manic or mixed episodes, including most currently used atypical antipsychotics, few have robust empirical data supportive of efficacy for acute bipolar depression. To date, 2 atypical antipsychotics have received regulatory approval for treatment of bipolar depression. Quetiapine is approved as monotherapy in the United States and European Union for treatment of depressive episodes associated with bipolar disorder [8] and an olanzapine-fluoxetine combination is approved in the United States for the same indication [9]. The adverse events, such as sedation and weight gain, associated with these drugs and the fact that not every patient responds equally well to treatment underscore the need to investigate additional treatment options.

Asenapine is an antipsychotic with a unique pharmacologic profile [10] indicated in the United States in adults for treatment of schizophrenia and as monotherapy or adjunctive therapy with lithium or valproate in the treatment of manic or mixed episodes associated with bipolar I disorder [11]. Asenapine is indicated in the European Union for the treatment of moderate to severe manic episodes associated with bipolar I disorder [12]. The multireceptor pharmacologic profile of asenapine includes antagonism at serotonergic 5-HT_2A _and adrenergic α_2 _receptors [10], suggesting that it may effectively treat depressive symptoms. The potential efficacy of asenapine against depressive symptoms is supported by preclinical findings in animal models [13].

In a pair of randomized placebo- and olanzapine-controlled 3-week trials enrolling patients with bipolar I disorder experiencing a current manic or mixed episode, asenapine demonstrated efficacy superior to placebo as early as day 2 in the treatment of acute mania; the active comparator in those studies (olanzapine) also demonstrated superiority over placebo [14,15]. In a 9-week extension of these trials, asenapine met criteria for noninferiority to olanzapine in the treatment of mania [16]. In a subsequent 40-week extension designed to assess long-term safety and tolerability, asenapine was well tolerated and maintained efficacy at a level comparable to olanzapine [17].

The current report describes an exploratory post hoc analysis of the 2 aforementioned 3-week monotherapy trials [14,15] undertaken to explore the effects of asenapine versus placebo on depressive symptoms in bipolar I patients experiencing acute manic or mixed episodes. Differences in the effects of asenapine versus olanzapine, the active control from these studies, were also assessed.

## Methods

### Study design

Data from 2 multinational, 3-week, randomized, flexible-dose, placebo- and olanzapine-controlled trials (NCT00159744; NCT00159796) were included. Each study was conducted in accordance with the principles of Good Clinical Practice and was approved by the appropriate institutional review boards and regulatory agencies. The study design and patient populations have been previously described [14,15]. In brief, the trials were conducted in 10 countries (United States, India, Russia, Ukraine, South Korea, Bulgaria, the Philippines, Romania, Turkey, and Malaysia). Each study included adult patients with a current *Diagnostic and Statistical Manual of Mental Disorders, Fourth Edition *diagnosis of manic or mixed episodes of bipolar I disorder. Included patients were required to have a Young Mania Rating Scale total score ≥20 at screening and baseline, a current manic or mixed bipolar I episode that began ≤3 months before screening, and a documented history of >1 moderate to severe manic or mixed episode, with or without psychotic features. Although limited doses of specific benzodiazepines and sleep medications were allowed during treatment week 1, all other psychotropic medications, including antidepressants, mood stabilizers, and St. John's wort, were prohibited [14,15].

### Treatment

After single-blind placebo run-in periods of ≤7 days, patients were randomly assigned to 3 weeks of sublingual asenapine (10 mg twice daily [BID] on day 1, flexible-dose 5 or 10 mg BID thereafter), placebo, or oral olanzapine (15 mg once daily [QD] on day 1, flexible-dose 5-20 mg QD thereafter) in a 2:1:2 ratio.

### Post hoc assessment of depressive symptoms

For these analyses, patients were divided into 3 depression-related populations, each of which is considered to denote clinically-relevant symptoms of depression:

• Baseline Montgomery-Asberg Depression Rating Scale (MADRS) total score ≥20

• Baseline Clinical Global Impression for Bipolar Disorder-Depression severity scale (CGI-BP-D) severity score ≥4

• Baseline diagnosis of a mixed episode

Change from baseline on the above scales was evaluated, as was the incidence of depression remission (ie, percentage of patients with MADRS total score ≤12) for each category on days 7 and 21.

In the primary trials, depression severity was assessed using the MADRS, the Positive and Negative Syndrome Scale (PANSS) Marder anxiety/depression factor, and the CGI-BP-D scale. MADRS and PANSS Marder anxiety/depression factor assessments were made on days 1, 7, and 21; the CGI-BP-D was administered at days 1, 2, 4, 7, 14, and 21. Baseline values were the last non-missing assessments on or prior to day 1 (randomization).

### Statistical analysis

Post hoc analyses were conducted for observed cases data on selected visits, as well as study Endpoint/Day 21 (using last observation carried forward [LOCF] if missing data occurred), for each data set.

Data from patients in each of the 2 studies were pooled for analysis; demographics and baseline MADRS and CGI-BP-D scores were balanced between treatment groups. Statistical analyses were conducted using an analysis of covariance on observed cases, with baseline values used as covariates; neither study nor the interaction of study × treatment effect were included as factors because no significant differences were found between studies. For continuous measures (MADRS, CGI-BP-D, and PANSS Marder anxiety/depression factor), comparisons were made for asenapine versus placebo, olanzapine versus placebo, and asenapine versus olanzapine on treatment days 7 and 21 using the difference in least squares (LS) mean change from baseline. Within-subject mean changes from baseline on days 7 and 21 were assessed using *t*-tests. Remission rate comparisons were made using Pearson chi-square tests. All statistical tests were 2-tailed, with statistical significance set at *P *< 0.05 (trends are reported if the *P*-values ranged from 0.05-0.09). No adjustments were made for multiple comparisons.

Data are presented in Tables 1 and 2 as the arithmetic mean ± SD and in all figures as the adjusted LS mean ± SE; *P*-values are based on the LS mean differences for between-group comparisons and arithmetic mean differences for within-subject changes.

## Results

### Study populations

The total number of randomized patients from the primary studies [14,15] included in the post hoc analyses and their baseline demographic and clinical characteristics are presented in Table 1. Of the 977 randomized patients in the primary studies, 212 (22%) met post hoc criteria for depression-related symptoms (MADRS ≥20 or CGI-BP-D ≥4) at baseline and 302 (31%) had a mixed episode at baseline; 90 (9.2%) met criteria for MADRS ≥20 or CGI-BP-D ≥4. Across groups, the percentages of patients meeting post hoc criteria for depression-related symptoms (MADRS ≥20 or CGI-BP-D ≥4) at baseline were 19% (72/379) for asenapine (MADRS ≥20 and CGI-BP-D ≥4; 32 [8.4%]), 24% (49/202) for placebo (MADRS ≥20 and CGI-BP-D ≥4; 21 [10.4%]), and 23% (91/396) for olanzapine (MADRS ≥20 and CGI-BP-D ≥4; 37 [9.3%]); for mixed episodes the percentages were 29% (111/379) for asenapine, 33% (67/202) for placebo, and 31% (124/396) for olanzapine.

**Table 1:** Demographics, clinical characteristics, and disposition

	Asenapine (n = 379)*	Placebo (n = 202)*	Olanzapine (n = 396)*
Patient populations, n			
Patients with mixed episodes^†^	111	67	124
Patients with MADRS total score ≥20^‡^	45	33	54
Patients with CGI-BP-D severity score ≥4^§^	59	37	74
Men, n (%)			
Patients with mixed episodes^†^	63 (56.8)	30 (44.8)	70 (56.5)
Patients with MADRS total score≥20^‡^	22 (48.9)	16 (48.5)	26 (48.1)
Patients with CGI-BP-D severity score ≥4^§^	31 (52.5)	18 (48.6)	37 (50)
Mean ± SD age, y			
Patients with mixed episodes^†^	38.3 ± 11.2	39.5 ± 12.5	38.8 ± 10.4
Patients with MADRS total score ≥20^‡^	38.3 ± 11.5	41.2 ± 11.6	39.5 ± 11.1
Patients with CGI-BP-D severity score ≥4^§^	39.4 ± 11.8	36.9 ± 12.7	39.6 ± 9.7
Mean ± SD daily dose, mg			
Patients with mixed episodes^†^	18.2 ± 2.8	-	15.6 ± 2.3
Patients with MADRS total score ≥20^‡^	18.3 ± 2.7	-	16.3 ± 2.5
Patients with CGI-BP-D severity score ≥4^§^	17.9 ± 2.6	-	15.9 ± 2.5
Mean ± SD MADRS total score			
Patients with mixed episodes^†^	16.7 ± 6.3	18.8 ± 7.3	16.9 ± 6.9
Patients with MADRS total score ≥20^‡^	24.4 ± 3.5	25.8 ± 4.7	24.7 ± 4.4
Patients with CGI-BP-D severity score ≥4^§^	20.2 ± 6.9	22.2 ± 7.5	19.7 ± 7.2
Mean ± SD CGI-BP-D severity score			
Patients with mixed episodes^†^	3.1 ± 1.3	3.4 ± 1.1	3.2 ± 1.1
Patients with MADRS total score ≥20^‡^	3.9 ± 0.9	3.8 ± 0.9	3.8 ± 0.8
Patients with CGI-BP-D severity score ≥4^§^	4.4 ± 0.6	4.3 ± 0.5	4.2 ± 0.4
Discontinuations, n (%)			
Patients with mixed episodes,^† ^overall	44 (39.6)	24 (35.8)	31 (25.0)
Adverse events	12 (10.8)	1 (1.5)	7 (5.6)
Lack of efficacy	8 (7.2)	8 (11.9)	7 (5.6)
Lost to follow-up	2 (1.8)	1 (1.5)	6 (4.8)
Withdrew consent	20 (18.0)	12 (17.9)	11 (8.9)
Other	2 (1.8)	2 (3.0)	0 (0)
Patients with MADRS total score ≥20,^‡ ^overall	19 (42.2)	11 (33.3)	13 (24.1)
Adverse events	6 (13.3)	1 (3.0)	2 (3.7)
Lack of efficacy	2 (4.4)	5 (15.2)	7 (13.0)
Lost to follow-up	2 (4.4)	0 (0)	1 (1.9)
Withdrew consent	8 (17.8)	4 (12.1)	3 (5.6)
Other	1 (2.2)	1 (3.0)	0 (0)
Patients with CGI-BP-D severity score ≥4,^§ ^overall	26 (44.1)	14 (37.8)	19 (25.7)
Adverse events	6 (10.2)	1 (2.7)	4 (5.4)
Lack of efficacy	4 (6.8)	4 (10.8)	8 (10.8)
Lost to follow-up	1 (1.7)	1 (2.7)	3 (4.1)
Withdrew consent	13 (22.0)	7 (18.9)	4 (5.4)
other	2 (3.4)	1 (2.7)	0 (0)

CGI-BP-D = Clinical Global Impression for Bipolar Disorder-Depression scale; MADRS = Montgomery-Asberg Depression Rating Scale.

*Total number of patients in the randomized treatment group in the original studies.

^†^Based on diagnosis at baseline (not post hoc assessment of MADRS or CGI-BP-D score)

^‡^Data represent patients with a MADRS total score ≥20 regardless of baseline CGI-BP-D severity score.

**^§^**Data represent patients with a CGI-BP-D severity score ≥4 at baseline regardless of baseline MADRS total score.

Baseline demographic characteristics were generally comparable across depression-related categories and treatment groups (Table 1). In patients with mixed episodes, the percentage of men in the placebo group was slightly lower than in the asenapine or olanzapine groups. The MADRS and CGI-BP-D severity scores were comparable across groups within each depression-related category. Patients experiencing mixed episodes had the lowest MADRS total and CGI-BP-D severity scores at baseline compared with those in other depression-related categories (Table 1 ).

The most common reasons for discontinuation across all depression-related categories were adverse events and withdrawn consent with asenapine, lack of efficacy and withdrawn consent with placebo, and lack of efficacy with olanzapine (Table 1).

### Efficacy

#### Montgomery-Asberg Depression Rating Scale total score

**Figure 1:**
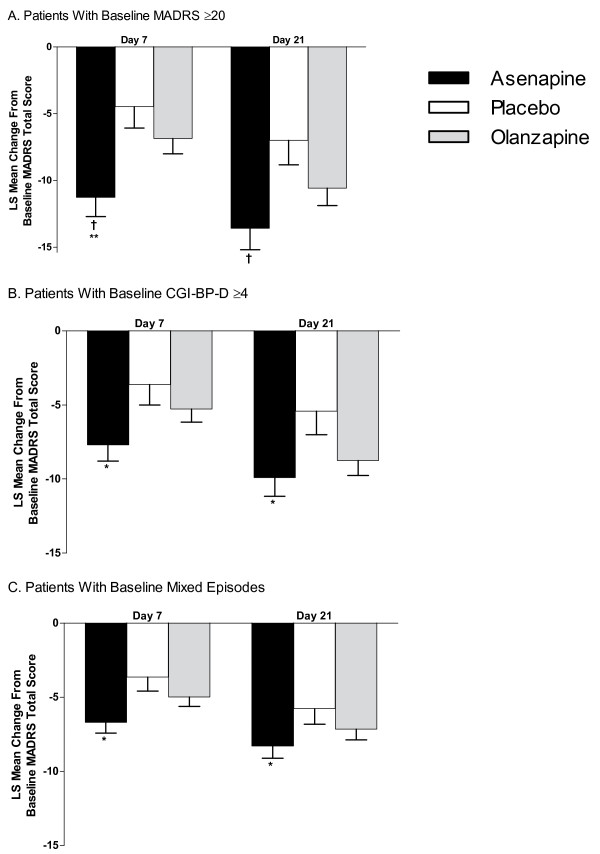
**Least Squares (LS) Mean Changes in Baseline MADRS Total Score**. (A) Patients with baseline MADRS total scores ≥20; (B) patients with baseline CGI-BP-D severity scores ≥4; (C) patients with a mixed episode at baseline. CGI-BP-D = Clinical Global Impression for Bipolar Disorder-Depression; MADRS = Montgomery-Asberg Depression Rating Scale. Error bars represent SE. *P < 0.05; ^†^*P *≤ 0.01 vs placebo. **P < 0.05 vs olanzapine.

In patients with baseline MADRS total scores ≥20, LS mean ± SE changes from baseline in MADRS total score with asenapine were significantly greater than placebo on days 7 (-11.3 ± 1.5 vs -4.5 ± 1.6; *P *= 0.002) and 21 (-13.6 ± 1.6 vs-7.0 ± 1.8; *P *= 0.009) and were greater than olanzapine on day 7 (-11.3 ± 1.5 vs -6.9 ± 1.2; *P *= 0.020). Change from baseline MADRS total score with olanzapine was not statistically different from placebo on day 7 (-6.9 ± 1.2 vs -4.5 ± 1.6; *P *= 0.231) or 21 (-10.6 ± 1.3 vs-7.0 ± 1.8; *P *= 0.121) (Figure 1A).

In patients with baseline CGI-BP-D severity scores ≥4, LS mean ± SE changes in MADRS total score with asenapine were significantly greater than placebo on days 7 (-7.7 ± 1.1 vs -3.6 ± 1.4; *P *= 0.023) and 21 (-9.9 ± 1.3 vs -5.4 ± 1.6; *P *= 0.030), with the difference from olanzapine showing a trend towards statistical significance on day 7 (-7.7 ± 1.1 vs -5.3 ± 0.9; *P *= 0.088). Change from baseline in MADRS total score with olanzapine was not statistically different from placebo at day 7 (-5.3 ± 0.9 vs -3.6 ± 1.4; *P *= 0.314), but it showed a trend towards statistical significance on day 21 (-8.8 ± 1.0 vs -5.4 ± 1.6; *P *= 0.084) (Figure 1B).

In patients with a mixed episode at baseline, LS mean ± SE changes in MADRS total score were significantly greater with asenapine than placebo on days 7 (-6.7 ± 0.7 vs -3.6 ± 1.0; *P *= 0.011) and 21 (-8.5 ± 0.8 vs -5.8 ± 1.1; *P *= 0.040), with the difference from olanzapine showing a trend towards statistical significance on day 7 (-6.7 ± 0.7 vs -5.0 ± 0.7; *P *= 0.076). Change from baseline in MADRS total score with olanzapine was not statistically different from placebo on days 7 (-5.0 ± 0.7 vs -3.6 ± 1.0; *P *= 0.244) or 21 (-7.2 ± 0.7 vs -5.8 ± 1.1; *P *= 0.269) (Figure 1C).

Mean ± SD changes from baseline in MADRS total score are summarized in Table 2. In all treatment groups and across all depression-related categories, within-subject changes from baseline on days 7 and 21 were statistically significant.

**Table 2:** Summary of mean changes from baseline in depressive symptoms for randomized patients

		Asenapine		Placebo		Olanzapine	
		
		Mean ± SD	*P *value	Mean ± SD	*P *value	Mean ± SD	*P *value
MADRS total score							
Patients with mixed episodes at baseline	Baseline	16.7 ± 6.3		18.8 ± 7.3		16.9 ± 6.9	
	Change at day 7	-6.3 ± 6.5	<0.0001	-4.4 ± 8.0	0.0011	-4.9 ± 5.3	<0.0001
	Change at day 21	-8.2 ± 7.6	<0.0001	-7.1 ± 8.2	<0.0001	-6.8 ± 7.0	<0.0001
Patients with MADRS total score ≥20 at baseline*	Baseline	24.4 ± 3.5		25.8 ± 4.7		24.7 ± 4.4	
	Change at day 7	-11.0 ± 7.6	<0.0001	-4.7 ± 9.5	0.0255	-6.9 ± 6.7	<0.0001
	Change at day 21	-12.9 ± 8.6	<0.0001	-8.4 ± 9.6	0.0007	-10.3 ± 8.8	<0.0001
Patients with CGI-BP-D severity score ≥4 at baseline^†^	Baseline	20.2 ± 6.9		22.2 ± 7.5		19.7 ± 7.2	
	Change at day 7	-7.5 ± 8.3	<0.0001	-4.1 ± 7.8	0.0188	-5.5 ± 5.2	<0.0001
	Change at day 21	-9.8 ± 8.6	<0.0001	-6.9 ± 10.7	0.0064	-8.2 ± 6.5	<0.0001
CGI-BP-D severity score							
Patients with mixed episodes at baseline	Baseline	3.1 ± 1.3		3.4 ± 1.1		3.2 ± 1.1	
	Change at day 7	-0.6 ± 0.9	<0.0001	-0.4 ± 1.1	0.0377	-0.7 ± 0.8	<0.0001
	Change at day 21	-1.0 ± 1.3	<0.0001	-0.8 ± 1.3	0.0004	-0.9 ± 1.1	<0.0001
Patients with MADRS total scores≥20 at baseline*	Baseline	3.9 ± 0.9		3.8 ± 0.9		3.8 ± 0.8	
	Change at day 7	-1.0 ± 1.0	<0.0001	-0.3 ± 1.2	0.1754	-0.8 ± 0.7	<0.0001
	Change at day 21	-1.5 ± 1.4	<0.0001	-0.7 ± 1.2	0.0228	-1.2 ± 1.2	<0.0001
Patients with CGI-BP-D severity score ≥4 at baseline^†^	Baseline	4.4 ± 0.6		4.3 ± 0.5		4.2 ± 0.4	
	Change at day 7	-1.2 ± 0.8	<0.0001	-0.6 ± 1.1	0.0127	-1.0 ± 0.9	<0.0001
	Change at day 21	-1.7 ± 1.2	<0.0001	-1.2 ± 1.1	0.0001	-1.6 ± 1.1	<0.0001
PANSS Marder anxiety/depression factor score							
Patients with mixed episodes at baseline	Baseline	12.9 ± 3.6		13.5 ± 3.5		12.6 ± 3.6	
	Change at day 7	-2.2 ± 3.5	<0.0001	-1.5 ± 3.6	0.0125	-1.7 ± 2.7	<0.0001
	Change at day 21	-3.4 ± 3.7	<0.0001	-3.0 ± 2.9	<0.0001	-2.8 ± 3.0	<0.0001
Patients with MADRS total score ≥20 at baseline*	Baseline	14.4 ± 3.3		14.5 ± 2.7		14.7 ± 3.1	
	Change at day 7	-3.7 ± 3.6	<0.0001	-0.9 ± 2.4	0.0784	-1.8 ± 3.0	0.0004
	Change at day 21	-4.9 ± 4.2	<0.0001	-2.4 ± 2.4	0.0002	-3.4 ± 3.7	<0.0001
Patients with CGI-BP-D severity score ≥4 at baseline^†^	Baseline	13.8 ± 3.7		14.5 ± 2.9		13.5 ± 3.7	
	Change at day 7	-2.8 ± 3.3	<0.0001	-1.1 ± 2.2	0.0243	-1.5 ± 3.2	0.001
	Change at day 21	-3.2 ± 4.5	0.0003	-2.4 ± 2.8	0.0007	-3.2 ± 3.4	<0.0001

CGI-BP-D = Clinical Global Impression for Bipolar Disorder-Depression scale; MADRS = Montgomery-Asberg Depression Rating Scale; PANSS = Positive and Negative Syndrome Scale.

*P *values are based on a two-sided *t*-test of within-subject mean changes from baseline.

*Data represent patients with a MADRS total score ≥20 regardless of baseline CGI-BP-D severity score.

^†^Data represent patients with a CGI-BP-D severity score ≥4 at baseline regardless of baseline MADRS total score.

#### Montgomery-Asberg Depression Rating Scale-based remission rates

**Figure 2:**
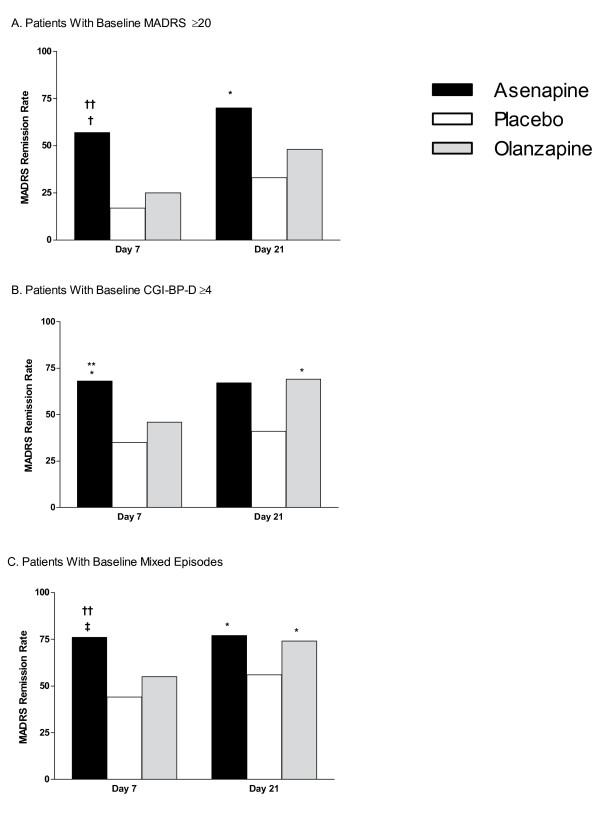
**MADRS Remission Rate**. (A) Patients with baseline MADRS total scores ≥20; (B) patients with baseline CGI-BP-D severity scores ≥4; (C) patients with a mixed episode at baseline. CGI-BP-D = Clinical Global Impression for Bipolar Disorder-Depression scale; MADRS = Montgomery-Asberg Depression Rating Scale. *P < 0.05; ^†^*P *≤ 0.01; ^‡^*P *≤ 0.001 vs placebo. **P < 0.05; ^††^*P *≤ 0.01 vs olanzapine.

In patients with baseline MADRS total scores ≥20, MADRS remission rates (defined as MADRS total score ≤12) with asenapine were significantly greater than placebo on days 7 (57% vs 17%; *P *= 0.004) and 21 (70% vs 33%; *P *= 0.012); remission rate with asenapine on day 7 was significantly greater than olanzapine (57% vs 25%; *P *= 0.006) and showed a trend towards statistical significance on day 21 (70% vs 48%; *P *= 0.066). Remission rates with olanzapine on days 7 (25%) and 21 (48%) were not statistically different from placebo (*P *= 0.478 and *P *= 0.288, respectively; Figure 2A).

In patients with baseline CGI-BP-D severity score ≥4, MADRS remission rates with asenapine were significantly greater than placebo on day 7 (68% vs 35%; *P *= 0.014) and showed a trend towards statistical significance on day 21 (68% vs 41%; *P *= 0.05); the remission rate with asenapine on day 7 was significantly greater than olanzapine (68% vs 45%; *P *= 0.031). MADRS remission rates with olanzapine were not significantly different from placebo on day 7 (45% vs 35%; *P *= 0.423) but was significantly greater on day 21 (69% vs 41%; *P *= 0.027) (Figure 2B).

In patients with a mixed episode at baseline, MADRS remission rates with asenapine were significantly greater than placebo on days 7 (76% vs 44%; *P *< 0.001) and 21 (78% vs 56%; *P *= 0.019); the remission rate with asenapine on day 7 was significantly greater than olanzapine (76% vs 55%; *P *= 0.007). Remission rates with olanzapine were not significantly different from placebo on day 7 (55% vs 44%; *P *= 0.259) but was significantly higher with olanzapine on day 21 (74% vs 56%; *P *= 0.04) (Figure 2C).

#### Clinical Global Impression for Bipolar Disorder-Depression Severity Scale score

**Figure 3:**
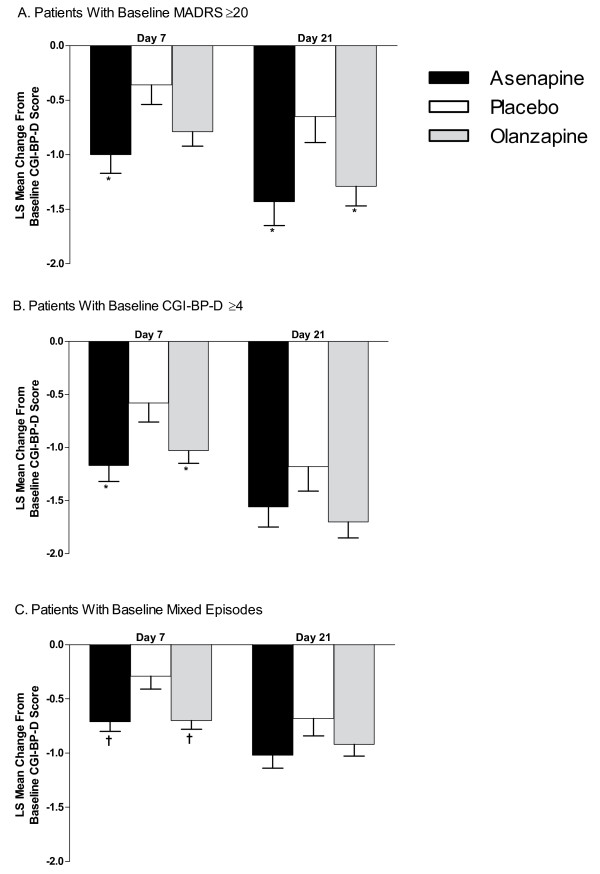
**Least Squares (LS) Mean Changes in Baseline CGI-BP-D Severity Score**. (A) Patients with baseline MADRS total scores ≥20; (B) patients with baseline CGI-BP-D severity scores ≥4; (C) patients with a mixed episode at baseline. CGI-BP-D = Clinical Global Impression for Bipolar Disorder-Depression scale; MADRS = Montgomery-Asberg Depression Rating Scale. Error bars represent SE. *P < 0.05; ^†^*P *≤ 0.01 vs placebo.

In patients with baseline MADRS total scores ≥20, LS mean ± SE changes from baseline in CGI-BP-D severity scores with asenapine were significantly greater than placebo on days 7 (-1.0 ± 0.2 vs -0.4 ± 0.2; *P *= 0.011) and 21 (-1.4 ± 02 vs -0.7 ± 0.2; *P *= 0.020) but did not differ statistically from olanzapine on either day 7 (*P *= 0.320) or 21 (*P *= 0.622). Changes with olanzapine versus placebo showed a trend towards statistical significance on day 7 (-0.8 ± 0.1 vs -0.4 ± 0.2; *P *= 0.062) and were significantly greater versus placebo on day 21 (-1.3 ± 0.2 vs -0.7 ± 0.2; *P *= 0.038) (Figure 3A).

In patients with baseline CGI-BP-D severity scores ≥4, LS mean ± SE changes in CGI-BP-D severity scores with asenapine were significantly greater than placebo on day 7 (-1.2 ± 0.2 vs -0.6 ± 0.2; *P *= 0.015) but not day 21 (-1.6 ± 0.2 vs -1.2 ± 0.23; *P *= 0.194) and did not differ statistically from olanzapine on either day 7 (*P *= 0.463) or 21 (*P *= 0.572). Changes with olanzapine were significantly greater than placebo on day 7 (-1.0 ± 0.1 vs -0.6 ± 0.2; *P *= 0.047) and showed a trend towards statistical significance on day 21 (-1.7 ± 0.2 vs -1.2 ± 0.2; *P *= 0.057) (Figure 3B).

In patients with a mixed episode at baseline, LS mean ± SE changes in CGI-BP-D severity score with asenapine were significantly greater than placebo on day 7 (-0.7 ± 0.1 vs -0.3 ± 0.1; *P *= 0.008) and approached significance on day 21 (-1.0 ± 0.1 vs -0.7 ± 0.2; *P *= 0.089); asenapine and olanzapine did not differ on either day 7 (*P *= 0.968) or 21 (*P *= 0.543). Changes with olanzapine were significantly greater than placebo on day 7 (-0.7 ± 0.1 vs -0.3 ± 0.1; *P *= 0.006) but not on day 21 (-0.9 ± 0.1 vs -0.7 ± 0.2; *P *= 0.203) (Figure 3C).

Mean ± SD changes from baseline CGI-BP-D severity scores are summarized in Table 2. Within-subject changes from baseline on days 7 and 21 were statistically significant in all treatment groups and across all depression-related categories, with 1 exception. Change in CGI-BP-D score on day 7 in patients with MADRS ≥20 treated with placebo was not statistically significant.

#### Positive and Negative Syndrome Scale Marder Anxiety/Depression Factor score

**Figure 4:**
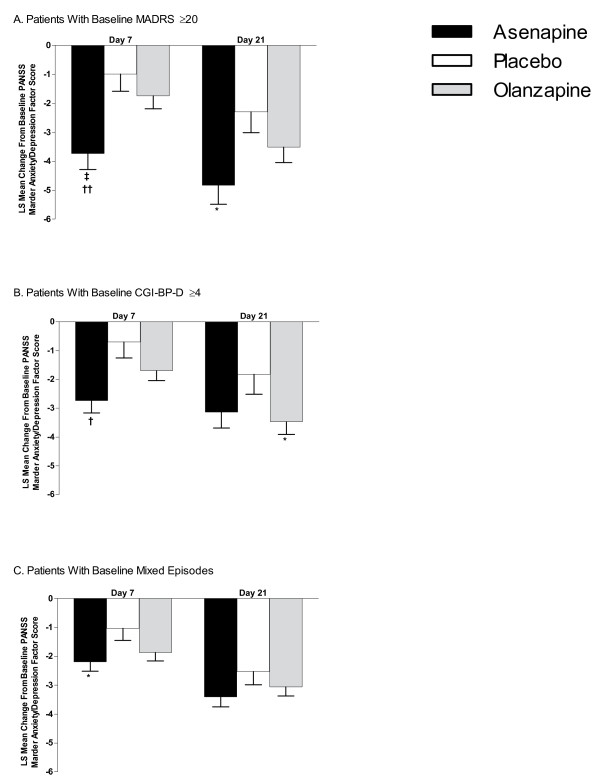
**Least Squares (LS) Mean Changes in Baseline PANSS Marder Anxiety/Depression Factor Score**. (A) Patients with baseline MADRS total scores ≥20; (B) patients with baseline CGI-BP-D severity scores ≥4; (C) patients with a mixed episode at baseline. CGI-BP-D = Clinical Global Impression for Bipolar Disorder-Depression scale; MADRS = Montgomery-Asberg Depression Rating Scale; PANSS = Positive and Negative Syndrome Scale. Error bars represent SE. *P < 0.05; ^†^*P *≤ 0.01; ^‡^*P *≤ 0.001 vs placebo. ^††^*P *≤ 0.01 vs olanzapine.

In patients with baseline MADRS total scores ≥20, LS mean ± SE changes from baseline in PANSS Marder anxiety/depression factor scores with asenapine were significantly greater than placebo on days 7 (-3.7 ± 0.6 vs -1.0 ± 0.6; *P *= 0.001) and 21 (-4.8 ± 0.7 vs -2.3 ± 0.7; *P *= 0.011) and greater than olanzapine on day 7 (-3.7 ± 0.6 vs -1.7 ± 0.4; *P *= 0.006). Changes with olanzapine (-1.7 ± 0.4 on day 7 and -3.5 ± 0.5 on day 21) did not statistically differ from placebo (*P *= 0.310 and 0.179; Figure 4A).

In patients with baseline CGI-BP-D severity scores ≥4, LS mean ± SE changes in PANSS Marder anxiety/depression factor scores with asenapine were significantly greater than placebo on day 7 (-2.7 ± 0.4 vs -0.7 ± 0.6; *P *= 0.005) but not day 21 (-3.1 ± 0.6 vs -1.8 ± 0.7; *P *= 0.139) and showed a trend towards statistical significance versus olanzapine on day 7 (-2.7 ± 0.4 vs -1.7 ± 0.4; *P *= 0.066) but not on day 21 (-3.1 ± 0.6 vs -3.5 ± 0.4; *P *= 0.647). Changes with olanzapine were not significantly different from placebo on day 7 (-1.7 ± 0.4 vs -0.7 ± 0.6; *P *= 0.133) but were significantly greater on day 21 (-3.5 0.4 vs -1.8 ± 0.7; *P *= 0.048) (Figure 4B).

In patients with a mixed episode, LS mean ± SE changes in PANSS Marder anxiety/depression scores with asenapine were significantly greater than placebo on day 7 (-2.2 ± 0.3 vs -1.0 ± 0.4; *P *= 0.031) but not day 21 (-3.4 ± 0.4 vs -2.5 ± 0.5; *P *= 0.129); changes with asenapine and olanzapine did not differ on either day 7 (*P *= 0.471) or 21 (*P *= 0.473). Changes with olanzapine (-1.9 ± 0.3 at day 7 and -3.1 ± 0.3 at day 21) were not statistically different from placebo (*P *= 0.105 and 0.331, respectively; Figure 4C).

Mean ± SD changes from baseline PANSS Marder anxiety/depression factor score are summarized in Table 2. With only 1 exception (change in PANSS Marder anxiety/depression factor score on day 7 in patients with MADRS ≥20 treated with placebo were not statistically significant), within-subject changes from baseline on days 7 and 21 were statistically significant in all treatment groups and across all depression-related categories.

## Discussion

In this exploratory post hoc analysis, asenapine was statistically superior to placebo in decreasing depressive symptoms in bipolar I disorder patients who were experiencing acute manic or mixed episodes and had clinically relevant depressive symptoms at baseline. Improvement was seen in all depression endpoints (change from baseline on MADRS total score, CGI-BP-D severity score, and PANSS Marder anxiety/depression factor score), as well as on MADRS remission rate. These results are based on analyses using observed cases at selected visits. To address the issue of missing data associated with early study discontinuation, study endpoint (using LOCF) were also reviewed. The LOCF results were in line with those reported from the observed case analysis.

The efficacy of asenapine in treating depressive symptoms is supported by in vitro and in vivo preclinical findings. Asenapine has a complex receptor signature, which includes combined antagonism at serotonergic (5-HT_2A _and 5-HT_2C _) and adrenergic (α_2_) receptors;[10] antagonism of these receptor subtypes has been linked to the amelioration of depressive symptoms[18,19]. Further, asenapine stimulates release of cortical dopamine, noradrenaline, and serotonin [20] and exerts an antidepressant-like effect in animal models[13].

Although various atypical antipsychotics have been evaluated for treatment of depressive episodes associated with bipolar disorder, the efficacy of these agents has varied substantially (see Table 3 for a summary of published results); currently only olanzapine in combination with fluoxetine and quetiapine monotherapy are approved by the US Food and Drug Administration for the treatment of bipolar depression [8,9]. In patients with bipolar depression, olanzapine alone and olanzapine in combination with fluoxetine significantly decreased MADRS total scores; placebo-corrected reductions over 3 to 8 weeks of treatment ranged from 3.1-4.4 points with olanzapine alone (versus 1.4-3.6 points for olanzapine alone in the studies included in this analysis) and 5.9-7.8 points when combined with fluoxetine [21]. In the current analysis, olanzapine also tended to improve depressive symptoms, but the olanzapine data appeared to be less consistent than those of asenapine. Additionally, asenapine was statistically superior to olanzapine in several instances (eg, day 7 change in MADRS and PANSS Marder anxiety/depression scores and MADRS remission rate). In the BipOLar DEpRession (ie, BOLDER) trials, quetiapine monotherapy significantly reduced MADRS total score compared with placebo, with placebo-corrected reductions in MADRS total scores of 4-5 points at week 3 and 4-6 points at week 8 reported in patients with bipolar I or II depression [22,23].

**Table 3:** Efficacy of selected antipsychotics for depressive symptoms in bipolar disorder: Summary of selected studies

		MADRS Total Score			CGI-BP-D	
				
	Study Design	Baseline	Change From Baseline	MADRS Remitters*	Baseline	Change From Baseline
**Asenapine**						

**(current post hoc analysis)**	Design: randomized, double-blind, placebo- and olanzapine-controlled study in bipolar I disorder patients experiencing manic or mixed episodes	*Baseline MADRS total score ≥20* Asenapine: 24.0 ± 3.5 Olanzapine: 25.0 ± 4.4 Placebo: 26.0 ± 4.7	LS mean ± SE at day 21 *Baseline MADRS total score ≥20* Asenapine: -13.6 ± 1.6 *(P = 0.009 *vs placebo) Olanzapine: -10.6 ± 1.3 Placebo: -7.0 ± 1.8	Percentage at day 21 *Baseline MADRS total score ≥20* Asenapine: 70% (*P *= 0.01 vs placebo) Olanzapine: 48% Placebo: 33%	*Baseline MADRS total score ≥20* Asenapine: 3.9 ± 0.9 Olanzapine: 3.8 ± 0.8 Placebo: 3.8 ± 0.9	LS mean ± SE at day 21 *Baseline MADRS total score ≥20* Asenapine: -1.4 ± 0.2 (*P *= 0.020 vs placebo) Olanzapine: -1.3 ± 0.2 (*P *= 0.038 vs placebo) Placebo: -0.7 ± 0.2
	Duration: 3 wk	*Baseline CGI-BP-D severity score ≥4* Asenapine: 20.0 ± 6.9 Olanzapine: 20.0 ± 7.2 Placebo: 22.0 ± 7.5	*Baseline CGI-BP-D severity score ≥4* Asenapine: -9.9 ± 1.3 (*P *= 0.030 vs placebo) Olanzapine: -8.8 ± 1.0 Placebo: -5.4 ± 1.6	*Baseline CGI-BP-D severity score ≥4* Asenapine: 67% Olanzapine: 69% (*P *= 0.026 vs placebo) Placebo: 41%	*Baseline CGI-BP-D severity score ≥4* Asenapine: 4.4 ± 0.6 Olanzapine: 4.2 ± 0.5 Placebo: 4.3 ± 0.5	*Baseline CGI-BP-D* *severity score ≥4* Asenapine: -1.6 ± 0.2 Olanzapine: -1.7 ± 0.2 Placebo: -1.2 ± 0.2
	Treatment: Asenapine 5-10 mg BID Olanzapine 5-20 mg QD Placebo	*Mixed episode at baseline* Asenapine: 17.0 ± 6.3 Olanzapine: 17.0 ± 6.9 Placebo: 19.0 ± 7.4	*Mixed episode at baseline* Asenapine: -8.5 ± 0.8 (*P *= 0.040 vs placebo) Olanzapine: -7.2 ± 0.7 Placebo: -5.8 ± 1.1	*Mixed episode at baseline* Asenapine: 77% (*P *= 0.026 vs placebo) Olanzapine: 74% (*P *= 0.036 vs placebo) Placebo: 56%	*Mixed episode at baseline* Asenapine: 3.1 ± 1.3 Olanzapine: 3.2 ± 1.1 Placebo: 3.4 ± 1.1	*Mixed episode at baseline* Asenapine: -1.0 ± 0.1 Olanzapine:-0.9 ± 0.1 Placebo:-0.7 ± 0.2

**Aripiprazole**						

Thase et al[22]	Design: randomized, double-blind, placebo-controlled study in bipolar I disorder patients experiencing a major depressive episode without psychotic features	Aripiprazole: 29.1 Placebo: 28.5	Adjusted mean ± SE at week 8 Not significant vs placebo (actual change not reported)	Percentage at week 8 Aripiprazole: 30% Placebo: 28%	Aripiprazole: 4.3 Placebo: 4.3	Adjusted mean ± SE at week 8 Not significant vs placebo (actual change not reported)
	Duration: 8 wk					
	Treatment: Aripiprazole 5-30 mg (n = 186) Placebo (n = 188)					
Thase et al[22]	Design: randomized, double-blind, placebo-controlled study in bipolar I disorder patients experiencing a major depressive episode without psychotic features	Aripiprazole: 29.6 Placebo: 29.4	Adjusted mean ± SE at week 8 Not significant vs placebo (actual change not reported)	Percentage at week 8 Aripiprazole: 26% Placebo: 29%	Aripiprazole: 4.4 Placebo: 4.5	Adjusted mean ± SE at week 8 Not significant vs placebo (actual change not reported)
	Duration: 8 wk					
	Treatment: Aripiprazole 5-30 mg (n = 187) Placebo (n = 188)					

**Olanzapine & olanzapine-fluoxetine**						

Tohen et al[18]	Design: randomized, double-blind, placebo-controlled study in bipolar I disorder patients with MADRS total score ≥20	Olanzapine: 32.6 Olanzapine-fluoxetine: 30.8 Placebo: 31.3	Mean ± SE at week 8 Olanzapine: -15.0 ± 0.7 (*P *= 0.002 vs placebo) Olanzapine-fluoxetine: -18.5 ± 1.3 (*P *< 0.001 vs placebo) Placebo: -11.9 ± 0.8	Percentage at week 8 Olanzapine: 33% (*P *= 0.02 vs placebo) Olanzapine-fluoxetine: 49% (*P *< 0.001 vs placebo) Placebo: 25%	Olanzapine: 4.9 ± 0.8 Olanzapine-fluoxetine: 4.8 ± 0.7 Placebo: 4.8 ± 0.8	Mean ± SE at week 8 Olanzapine: -1.6 ± 0.1 (*P *= 0.004 vs placebo) Olanzapine-fluoxetine: -2.2 ± 0.2 (*P *< 0.001 vs placebo) Placebo:-1.2 ± 0.1
	Duration: 8 wk					
	Treatment: Olanzapine 5-20 mg (n = 370) Olanzapine-fluoxetine 6-12 mg and 25-50 mg (n = 86) Placebo (n = 377)					

**Quetiapine**						

Thase et al[20]	Design: randomized, double-blind, placebo-controlled in bipolar I or II disorder patients experiencing a major depressive episode	Quetiapine 300 mg: 31.1 ± 5.7 Quetiapine 600 mg: 29.9 ± 5.6 Placebo: 29.6 ± 5.4	LS mean ± SE at last assessment Quetiapine 300 mg: -16.9 ± 1.0 (*P *< 0.001 vs placebo) Quetiapine 600 mg: -16.0 ± 1.0 (*P *= 0.001 vs placebo) Placebo: -11.9 ± 1.0	Percentage at last assessment Quetiapine 300 mg: 52% (*P *< 0.05 vs placebo) Quetiapine 600 mg: 52% (*P *< 0.01 vs placebo) Placebo: 37%	NA	NA
	Duration: 8 wk					
	Treatment: Quetiapine 300 mg (n = 172) Quetiapine 600 mg (n = 169) Placebo (n = 168)					
Calabrese et al[19]	Design: randomized, double-blind, placebo-controlled in bipolar I or II disorder patients experiencing a major depressive episode	Quetiapine 300 mg: 30.4 ± 5.0 Quetiapine 600 mg: 30.3 ± 5.3 Placebo: 30.6 ± 5.3	Mean at last assessment Quetiapine 300 mg: -16.4 (*P *< 0.001 vs placebo) Quetiapine 600 mg: -16.7 (*P *< 0.001 vs placebo) Placebo: -10.3	Percentage at last assessment Quetiapine 300 mg: 53% (*P *< 0.001 vs placebo) Quetiapine 600 mg: 53% (*P *< 0.001 vs placebo) Placebo: 28%	NA	NA
	Duration: 8 wk					
	Treatment: Quetiapine 300 mg (n = 181) Quetiapine 600 mg (n = 180) Placebo (n = 181)					

**Ziprasidone**						

Liebowitz et al[24]	Design: open-label in bipolar II disorder patients experiencing a major depressive episode	Ziprasidone: 28.5 ± 5.0	Mean change ± SD at week 8 Ziprasidone: 13.2 ± 9.0 (*P *< 0.0001 vs baseline)	NA	NA	NA
	Duration: 8 wk					
	Treatment: Ziprasidone 20 mg QD -60 mg BID (n = 30)					

BID = twice daily; CGI-BP-D = Clinical Global Impression for Bipolar Disorder-Depression scale; LS = least squares; MADRS = Montgomery-Asberg Depression Rating Scale; NA = not applicable; QD = once daily.

*For aripiprazole, defined as MADRS total score ≤8; for all others, defined as MADRS total score ≤12.

Despite being approved for adjunctive use in the treatment of major depressive disorder [24], aripiprazole was no more effective than placebo in alleviating depressive symptoms at endpoint in patients with bipolar I disorder [25]. Risperidone as an adjunct to mood stabilizer treatment was associated with a recovery rate of only 5% in an open-label trial of treatment-resistant patients with bipolar I or II disorder experiencing depressive episodes [26]. Ziprasidone was effective in the treatment of bipolar II disorder patients experiencing major depressive episodes in an open-label trial [27] and in treating depressive symptoms in a post hoc analysis of bipolar patients experiencing dysphoric mania [28]; however, reviews indicate that ziprasidone was not superior to placebo in controlled studies of patients with bipolar depression [6,29].

Although direct comparisons between this exploratory post hoc analysis and randomized clinical trials should be made cautiously, the placebo-corrected changes in MADRS total score in the current analysis (asenapine, 2.6-6.6 points; olanzapine, 1.4-3.6 points) are in the same range as those previously reported in patients with bipolar I or II depression receiving quetiapine or in patients with bipolar I depression receiving olanzapine/fluoxetine [21-23]. They are also within the range of values reported in a meta-analysis of controlled bipolar depression trials of quetiapine, olanzapine, and aripiprazole, which reported overall mean MADRS total score reductions of 3.91 points (95% CI, -5.55 to -2.26) versus placebo; this value increased to 4.90 points (95% CI, -6.21 to -3.59) when negative aripiprazole trials were excluded [30].

In this post hoc analysis, differential effects were observed among depression-related categories, with reductions in depressive symptoms being more robust in patients with baseline MADRS total scores ≥20 than in those with baseline CGI-BP-D severity scores of ≥4 or those experiencing a mixed episode. This variation might result from the rating scales used. Although a MADRS total score of 20 and CGI-BP-D severity score of 4 corresponds to moderate depressive symptoms [31,32], respectively, the CGI-BP-D may be less sensitive to change than the MADRS, reducing the ability to detect depressive symptom changes in patients with baseline CGI-BP-D severity score ≥4 (on a 7-point scale) versus in those with a baseline MADRS total scores ≥20 (on a 60-point scale). Comparisons with patients experiencing a mixed episode for the purposes of this post hoc analysis could also be problematic. Due to the higher overall level of variability in baseline depressive symptoms in patients with mixed episodes, the possibility of detecting statistically significant changes in this post hoc analysis may have been compromised.

## Conclusions

Depression is considered the predominant burden of bipolar disorder, with depressive states accounting for about 75% of the typical unwell time in bipolar I and II disorder [6]. Therefore, additional effective treatment options are needed for bipolar patients with depressive symptoms. In these exploratory post hoc analyses, asenapine reduced depressive symptoms in bipolar I disorder patients experiencing acute manic or mixed episodes with clinically-relevant depressive symptoms at baseline. These data suggest asenapine may be useful in the treatment of depressive episodes associated with bipolar disorder. However, the results of these analyses need to be interpreted in light of the fact that the primary study population was diagnosed with manic or mixed episodes rather than acute bipolar depression at the time of study entry. Furthermore, because these analyses were performed in a subset of patients from the original trials, the sample size for this post hoc analysis is small and not necessarily representative of the target population. Prospective controlled clinical trials in patients with bipolar depression are needed to definitively demonstrate the efficacy of asenapine in the treatment of depressive symptoms in bipolar disorder.

## Declaration of competing interests

Drs. Szegedi, Zhao, Nations, and Mackle are full-time employees of Merck. Dr. Panagides was an employee of Schering-Plough (formerly Organon), now Merck at the time this analysis was conducted. Dr. van Willigenburg was an employee of Schering-Plough (formerly Organon) at the time this research was conducted.

## Authors' contributions

AS was involved in the oversight of the trials, the analysis and interpretation of these post hoc analyses, and in the preparation and finalization of the manuscript. JP was involved in the design and oversight of the trials, the analysis and interpretation of these post hoc analyses, and in the preparation and finalization of the manuscript. AvW and KN were involved in the analysis and interpretation of these post hoc analyses and in the preparation and finalization of the manuscript. MM was involved in the design of processes and standards for data acquisition during the original trials, in the harmonization of protocol interpretation across sites involved in the original trials, made substantial contributions to the analysis and interpretation of data described within the publication, and contributed to the development and critical review of the intellectual content of the manuscript. JZ designed and conducted the statistical analyses described in the manuscript. All authors approved submission of the final version of the manuscript.

## Pre-publication history

The pre-publication history for this paper can be accessed here:

http://www.biomedcentral.com/1471-244X/11/101/prepub
